# The Effect of Three-Dimensional Stabilization Thread Design on Biomechanical Fixation and Osseointegration in Type IV Bone

**DOI:** 10.3390/biomimetics10060395

**Published:** 2025-06-12

**Authors:** Nicholas J. Iglesias, Vasudev Vivekanand Nayak, Arthur Castellano, Lukasz Witek, Bruno Martins de Souza, Edmara T. P. Bergamo, Ricky Almada, Blaire V. Slavin, Estevam A. Bonfante, Paulo G. Coelho

**Affiliations:** 1 DeWitt Daughtry Family Department of Surgery, University of Miami Miller School of Medicine, Miami, FL 33136, USA; 2 Department of Biochemistry and Molecular Biology, University of Miami Miller School of Medicine, Miami, FL 33136, USA; 3 Dr. John T. Macdonald Foundation Biomedical Nanotechnology Institute (BioNIUM), University of Miami, Miami, FL 33136, USA; 4 Mackenzie Evangelical School of Medicine Paraná, Curitiba 80730-000, PR, Brazil; 5 Federal University of Paraná, Curitiba 80060-000, PR, Brazil; 6 Biomaterials and Regenerative Biology Division, NYU College of Dentistry, New York, NY 10010, USA; 7 Department of Biomedical Engineering, NYU Tandon School of Engineering, Brooklyn, NY 11201, USA; 8 Hansjörg Wyss Department of Plastic Surgery, NYU Grossman School of Medicine, New York, NY 10016, USA; 9 Department of Oral and Maxillofacial Surgery, NYU College of Dentistry, New York, NY 10010, USA; 10 Laboratory of Biomaterials, Military Engineering Institute, Rio de Janeiro 22290-270, RJ, Brazil; 11 Department of Prosthodontics, NYU College of Dentistry, New York, NY 10010, USA; 12 University of Miami Miller School of Medicine, Miami, FL 33136, USA; 13 Department of Prosthodontics and Periodontology, Bauru School of Dentistry, University of São Paulo, Bauru 17012-901, SP, Brazil; 14 DeWitt Daughtry Family Department of Surgery, Division of Plastic Surgery, University of Miami Miller School of Medicine, Miami, FL 33136, USA

**Keywords:** insertion torque value, implant stability quotient, osseointegration, primary stability, nanoindentation, lateral loading

## Abstract

Achieving the appropriate primary stability for immediate or early loading in areas with low-density bone, such as the posterior maxilla, is challenging. A three-dimensional (3D) stabilization implant design featuring a tapered body with continuous cutting flutes along the length of the external thread form, with a combination of curved and linear geometric surfaces on the thread’s crest, has the capacity to enhance early biomechanical and osseointegration outcomes compared to implants with traditional buttressed thread profiles. Commercially available implants with a buttress thread design (TP), and an experimental implant that incorporated the 3D stabilization trimmed-thread design (TP 3DS) were used in this study. Six osteotomies were surgically created in the ilium of adult sheep (N = 14). Osteotomy sites were randomized to receive either the TP or TP 3DS implant to reduce site bias. Subjects were allowed to heal for either 3 or 12 weeks (N = 7 sheep/time point), after which samples were collected en bloc (including the implants and surrounding bone) and implants were either subjected to bench-top biomechanical testing (e.g., lateral loading), histological/histomorphometric analysis, or nanoindentation testing. Both implant designs yielded high insertion torque (ITV ≥ 30 N⋅cm) and implant stability quotient (ISQ ≥ 70) values, indicative of high primary stability. Qualitative histomorphological analysis revealed that the TP 3DS group exhibited a continuous bone–implant interface along the threaded region, in contrast to the TP group at the early, 3-week, healing time point. Furthermore, TP 3DS’s cutting flutes along the entire length of the implant permitted the distribution of autologous bone chips within the healing chambers. Histological evaluation at 12 weeks revealed an increase in woven bone containing a greater presence of lacunae within the healing chambers in both groups, consistent with an intramembranous-like healing pattern and absence of bone dieback. The TP 3DS macrogeometry yielded a ~66% increase in average lateral load during pushout testing at baseline (T = 0 weeks, *p* = 0.036) and significantly higher bone-to-implant contact (BIC) values at 3 weeks post-implantation (*p* = 0.006), relative to the traditional TP implant. In a low-density (Type IV) bone model, the TP 3DS implant demonstrated improved performance compared to the conventional TP, as evidenced by an increase in baseline lateral loading capacity and increased BIC during the early stages of osseointegration. These findings indicate that the modified implant configuration of the TP 3DS facilitates more favorable biomechanical integration and may promote more rapid and stable bone anchorage under compromised bone quality conditions. Therefore, such improvements could have important clinical implications for the success and longevity of dental implants placed in regions with low bone density.

## 1. Introduction

Osseointegration is defined as the apposition of bone with an implant, and the structural and functional connection at the bone–implant interface [1,2]. Implant stability, as a function of time, can be subcategorized into primary and secondary stability. Primary stability is defined as a mechanical phenomenon that is typically highest immediately following implant placement, owing to the mechanical compression of the implant body on the osteotomy walls [3,4,5]. On the other hand, secondary stability can be defined as the progressive increase in stability as a result of an in vivo response (new bone formation and remodeling) at the bone–implant interface [5]. However, if the implant lacks primary stability during the initial healing phase, the biological response may shift toward soft tissue formation at the interface, potentially resulting in fibrous encapsulation, and consequently, reduced secondary stability. As a result, achieving primary stability is considered critical for successful osseointegration outcomes [6]. Multiple studies have investigated the primary stability and success rates of endosteal implants, factors that influence the healing process, and overall clinical outcomes [7,8,9,10,11]. Notably, primary stability is influenced by implant design characteristics such as shape, thread geometry, and cutting-edge configuration. The underlying quantity and density of the host bone at the implantation site have also been shown to be contributing factors [7,8,9,10,11,12,13,14,15].

Cylindrical and tapered macrogeometries are commonly utilized dental implant designs [16]. Tapered implants, in particular, have been associated with improved primary stability, as evidenced by high insertion torque values (ITVs) and implant stability quotient (ISQ) values [16,17]. Thread geometry represents another key variable in implant design, with screw-type, plateau, and buttressed configurations being most commonly utilized [18]. The interaction between implant threads and the surrounding bone within the osteotomy site therefore plays a critical role in determining the mechanical stability at the bone–implant interface [19]. Specifically, implant macrogeometries that incorporate intentional void spaces—commonly referred to as healing chambers—have been shown to facilitate intramembranous ossification [19]. Bone formation within these healing chambers is characterized by chondrocyte and osteoblast ingrowth. This initial lamellar bone formation progresses swiftly and is subsequently followed by a remodeling phase, ultimately resulting in the development of mature lamellar bone [8].

However, achieving predictable osseointegration in low-density trabecular bone, such as that found in the posterior maxilla, remains a significant clinical challenge. The limited primary stability in this region is mainly attributed to the presence of thin cortical bone, poor trabecular bone quality, and reduced vertical bone height due to the proximity of the maxillary sinus [4]. Surgical techniques have been suggested to improve primary stability in this region, with the common clinical approach involving the preparation of an under-sized osteotomy [20]. However, this method may lead to adverse outcomes, including implant fixation failure, as it can induce microcracking at the osteotomy sites due to strain that exceeds the elastic limits of bone [21].

Similarly, variations in cutting flute designs have been suggested to modify the biomechanical strain on bone during implant insertion [22]. Different cutting flute geometries have been shown to influence implant stability, with traditional cutting flutes, particularly at the apical aspect of the implant, often being linked to a reduction in primary stability [23]. In this context, a three-dimensional (3D) stabilization implant design (TP 3DS, BioHorizons, Birmingham, AL, USA) integrates a tapered body with continuous cutting flutes along the entire length of the external thread [24,25]. The external thread presents a combination of curved and linear geometric surfaces across the crest [24,25]. This novel thread design aims to maximize retention forces, reduce friction during insertion, and minimize lateral movement under physiological loading [24,25,26].

Our previous study demonstrated that the TP 3DS reduces ITV and ISQ values, while enhancing resistance to lateral loading during the early stages of osseointegration relative to implants with conventional buttressed thread profiles with apically located cutting flute geometries [26]. However, the existing literature on the TP 3DS implants has primarily focused on biomechanical evaluations in pre-clinical bone models with higher cortical density (Type I) [26]. Given the variable turnover rate, increased vascularity, reduced density, and increased porosity of trabecular bone compared to cortical bone, further investigation on the impact of the TP 3DS geometry in this pre-clinical setting is warranted prior to clinical application [27]. Therefore, the aim of this study was to investigate the effects of the TP 3DS on biomechanical performance and osseointegration outcomes within a low bone density (Type IV) environment.

## 2. Materials and Methods

### 2.1. Implant Macrogeometries

**Figure 1:**
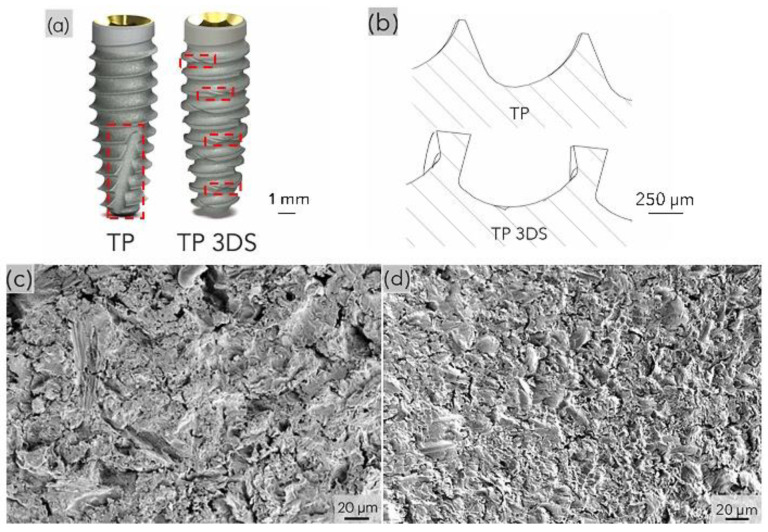
(**a**) Representative pictographic overviews of the implant macrogeometries, with dashed red boxes highlighting the location of the cutting flutes; (**b**) representative thread profiles; scanning electron microscopy of the (**c**) TP and (**d**) TP 3DS implant surfaces. Authors’ own work, adapted with permission [26], copyright 2025 Elsevier Ltd.

Two implant designs were selected: a commercially available implant featuring a conventional buttress thread design (TP: Tapered Pro, BioHorizons, Birmingham, AL, USA), and an experimental implant incorporating a 3D stabilization thread design (TP 3DS, BioHorizons, Birmingham, AL, USA) (Figure 1a) [24,25,26]. Both TP and TP 3DS implants were 9 mm in length, with a 4.2 mm diameter tapered body and a 3.5 mm diameter prosthetic connection [28]. In contrast to the standard buttress thread design with a single, large apical cutting flute (TP), the TP3DS implant incorporated a distinctive combination of curved and linear geometric surfaces, along with continuous cutting flutes extending along the full length of the external threads. Thread depth, pitch, and shape can be visualized in the schematic (Figure 1b) [26]. To isolate the effects of the cutting flute and thread configuration on biomechanical performance and osseointegration, both implant groups presented identical surface topographies (Figure 1c,d) [26].

### 2.2. Surgical Procedure

**Figure 2:**
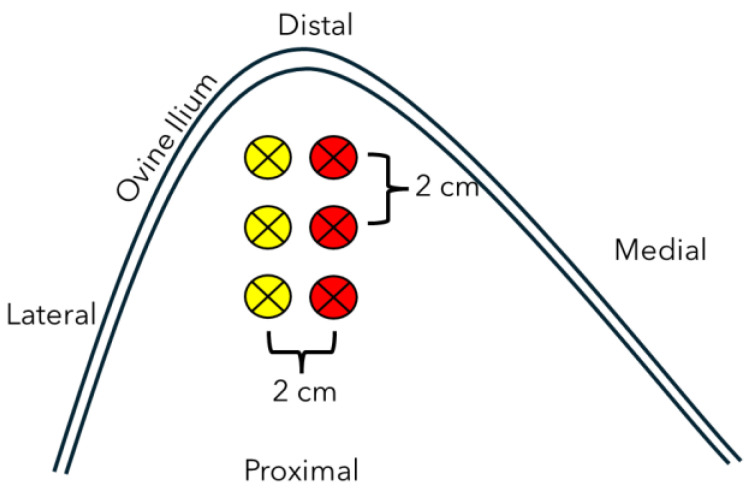
Representative schematic of osteotomy and implant placement within the ovine ilium. TP implants are shown in yellow and TP 3DS implants in red. Image not to scale.

The surgical procedure for this study received approval from the Institutional Animal Care and Use Committee at École Nationale Vétérinaire d’Alfort (Maisons-Alfort, Ile-de-France, France, file number: 13-011; notice number: 05/14/13-3). A total of 14 healthy adult sheep were obtained and allowed to acclimate for ~1 week before any surgical procedures. Prior to surgical intervention, general anesthesia was induced with sodium pentothal (15–20 mg/kg) in Normasol solution via injection into the jugular vein and maintained using 1.5–3% isoflurane in 50/50 O_2_/N_2_O, as needed. Prior to surgery, areas surrounding the hip were shaved and prepared in accordance with aseptic and sterile techniques. The skin was sharply incised, and subcutaneous tissues were dissected to gain access and expose a large area of the ovine iliac crest. Subsequently, six osteotomies were surgically prepared as per the manufacturer protocols: pilot drill (2 mm at 1500 rpm) and osteotomy development (2.5 mm, 3.2 mm and 3.7 mm at 1000 rpm) under continuous irrigation to reduce thermal osteonecrosis. Osteotomies were separated by approximately 2 cm. To reduce bias, each osteotomy site was randomized to receive the TP or TP 3DS implant. A representative schematic demonstrating implant placement is shown in Figure 2. As the implants were inserted into the osteotomies (clockwise rotation), ITV (N⋅cm) was measured using a digital torque wrench (Tohnichi, Tokyo, Japan) equipped with a 200 N⋅cm load cell. The immediate post-insertion ISQ was also measured using a SmartPeg™ attachment that was connected to a resonance frequency analysis instrument (Beacon, Osstell AB, Göteborg, Sweden). Subjects were allowed to heal for either 3 or 12 weeks and euthanized at the respective time points as per the approved protocol. The implants and surrounding bone were collected en bloc and the implants (n = 21 implants per group per time point) were equally distributed between bench-top biomechanical testing, histological/histomorphometric analysis, and nanoindentation testing.

### 2.3. Biomechanical Testing

Ex vivo samples were processed to size to be accommodated within a universal testing machine (Instron, Norwood, MA, USA). The universal testing machine was equipped with a ±1000 N load cell and a lateral force was applied such that the direction of loading was perpendicular to the implant’s longitudinal section at a rate of 1 mm/min. This loading direction was chosen to induce interfacial fractures at the bone–implant interface [26]. The peak lateral load (in N) was recorded for each implant. Baseline lateral load values (equivalent to T = 0 weeks) were obtained using implants (n = 6 per group per animal) inserted into previously unoperated, ex vivo segments of the ilium and underwent identical processing and lateral load testing.

### 2.4. Histological Analysis

**Figure 3:**
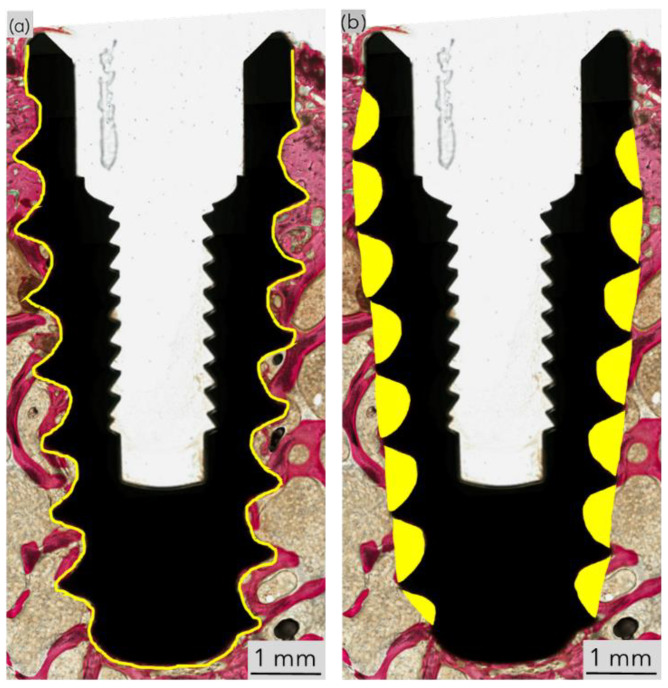
Representative histological section showing the (**a**) total implant perimeter (yellow spline) for measurement of BIC, and (**b**) total area of the implant threads (sections highlighted in yellow) for measurement of BAFO. Implant macrogeometry is shown in black and calcified tissue in red.

Sequentially, specimens were dehydrated in 70–100% ethanol (EtOH), immersed in methyl salicylate, and embedded in methacrylate-based resin. Samples were sliced along the longitudinal axis of the implant into ~300 μm thick sections with a low-speed precision wafering saw (Isomet 2000, Buehler Ltd., Lake Bluff, IL, USA). Slices were reduced to a final thickness of ~100 μm using progressively finer SiC abrasive sheets (400, 600, 800, and 1200 grit) on a rotary grinding machine (Metaserv 3000, Buehler, Lake Bluff, IL, USA) under copious irrigation. Slides were polished using a polishing solution (1 μm MicroPolish^TM^, Buehler, Lake Bluff, IL, USA) on a microfiber cloth for one minute. Slides were stained with Stevenel’s Blue and Van Gieson picrofuschin (SVG) and digitally scanned (Aperio CS2, Leica, Wetzlar, Germany) for quantitative histomorphometric analysis. ImageJ (version 1.54e, National Institutes of Health, Bethesda, MD, USA) was employed to assess bone-to-implant contact (BIC) and bone area fractional occupancy (BAFO). In brief, BIC was calculated as the ratio of the perimeter of the implant surface in direct contact with newly formed bone to the total implant perimeter; BAFO was calculated as the ratio of the bone area within the implant threads to the total area of the implant threads, expressed as percentages (illustrated in Figure 3). Histomorphometric analysis was performed by a single, blinded, and trained investigator [26].

### 2.5. Nanoindentation Testing

Histological slides were further polished using increasingly finer diamond-based suspensions (9 µm to 0.1 µm, Electron Microscopy Sciences, Hatfield, PA, USA) on a microfiber cloth to ensure a smooth surface and minimize unintended contact issues between the slide and the nano-indenter tip. Following the polishing steps, slides were sonicated for 3 min. Nine indentations (in a 3 × 3 grid in the x- and y-axes) were created at the bone–implant interface region within the healing chambers utilizing a Berkovich tip (Hysitron TI 950 Nano-indenter, Bruker, Billerica, MA, USA) with each indent spaced 10 µm apart. For each indentation, load–displacement curves were generated, and the in-built software was used to compute Young’s modulus and hardness (in GPa) of bone, as demonstrated earlier [26].

### 2.6. Statistical Analysis

A linear mixed model analysis of variance was employed (SPSS v29, IBM Corp., Armonk, NY, USA), specifically due to the nested within-subject observations. This approach accounted for random effects arising from animal variations and implant location within the ilium in the analysis of outcome variables. All analyses were carried out with fixed factors of time and group (TP vs. TP 3DS), with data presented as mean ± 95% confidence intervals (CIs) unless otherwise specified and *p* < 0.05 denoting statistical significance.

## 3. Results

### 3.1. Biomechanical Analysis

**Figure 4:**
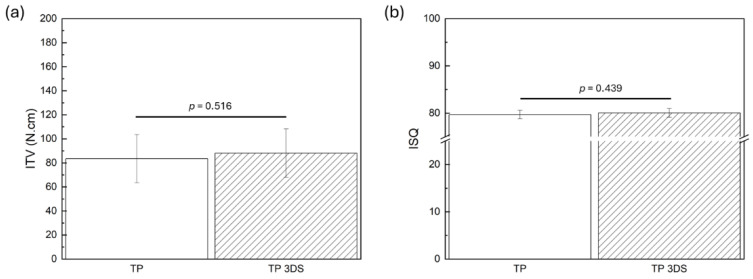
(**a**) Insertion torque value (ITV, Ncm) at T = 0 weeks, and (**b**) implant stability quotient (ISQ, measured on a unitless scale) at T = 0 weeks. *p* < 0.05 is statistically significant.

**Figure 5:**
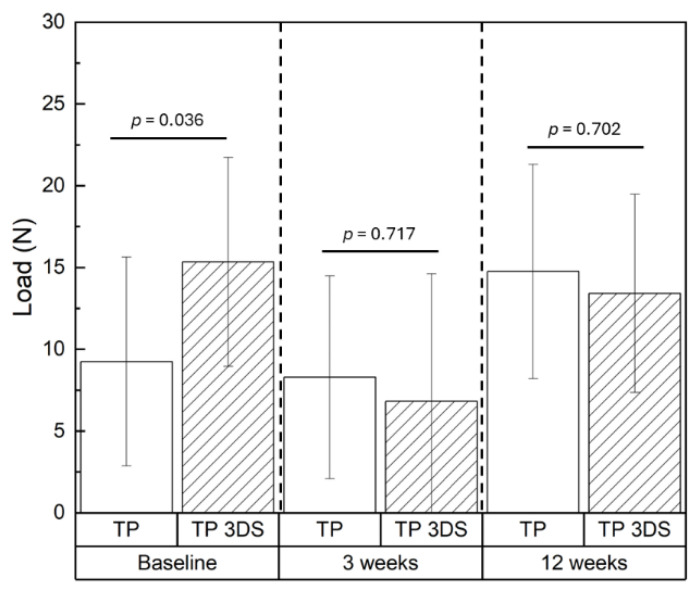
Lateral load (N) data compared between the TP and TP 3DS groups at the various time points of evaluation. Baseline corresponds to the T = 0-weeks. *p* < 0.05 is statistically significant.

The ITV (Figure 4a) and ISQ (Figure 4b) were statistically homogenous between TP and TP 3DS (*p* = 0.52 and *p* = 0.44, respectively). Pertaining to lateral load testing, TP 3DS implants demonstrated a significant (~66%) increase in average load values at baseline (T = 0 weeks) relative to their TP counterparts (mean ± standard deviation: 15.34 ± 8.84 N vs. 9.25 ± 7.05 N, respectively; *p* = 0.036) (Figure 5). However, no significant differences in load values between TP and TP 3DS groups were observed at 3 (*p* = 0.717) or 12 weeks (*p* = 0.702).

### 3.2. Qualitative Histological Evaluation

**Figure 6:**
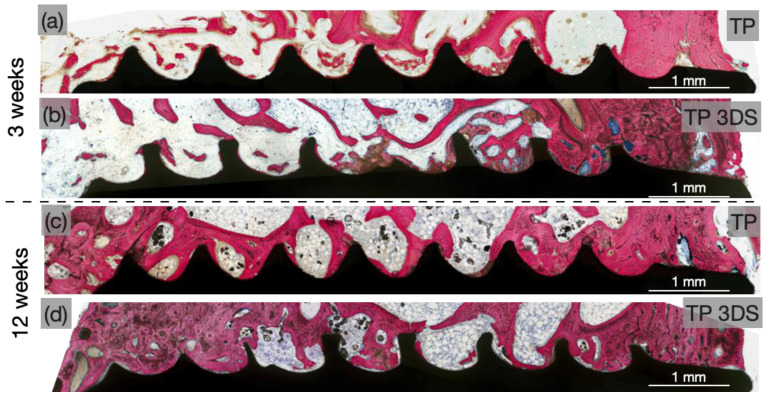
Representative histological overviews of TP and TP 3DS implants at (**a**,**b**) 3 and (**c**,**d**) 12 weeks post-implantation. Implant macrogeometry is shown in black and calcified tissue in red.

**Figure 7:**
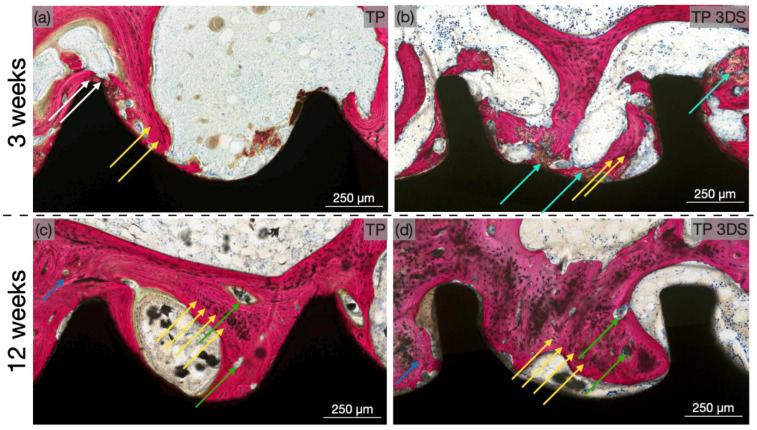
Representative high-magnification histomicrographs of TP and TP 3DS implants at (**a**,**b**) 3 and (**c**,**d**) 12 weeks post-implantation. Implant macrogeometry is shown in black and calcified tissue in red. The white arrow represents microcracks in bone, cyan arrows depict bone chips present within the implant healing chambers, green arrows identify sites of bone remodeling, and blue arrows highlight sites of lamellar bone growth. Yellow arrows depict lacunae within the healing chambers.

Qualitative evaluation of the histological micrographs revealed successful osseointegration of implants of both macrogeometries at 3 weeks (Figure 6a,b) and 12 weeks (Figure 6c,d). At 3 weeks, woven bone surrounded the apical regions of the implant threads across all groups, corresponding to sites of initial bone interlocking. Additionally, new bone formation was evident throughout the healing chambers within the trabecular region of the ilium. Furthermore, at 3 weeks, the TP 3DS group (Figure 6b) exhibited a more continuous bone–implant interface along the coronal aspect of the threaded region compared to the TP group (Figure 6a). At this time point, microcracks—indicative of bone exceeding its yield strength due to elevated stress—were observed, along with signs of early remodeling between the TP implant threads, likely resulting from compression-induced necrosis (Figure 7a). In contrast, TP 3DS exhibited cutting flutes extending along the entire implant length, facilitating the redistribution of autologous bone chips within the healing chambers (Figure 7b). Histological evaluation at 12 weeks revealed an increase in woven bone containing greater presence of lacunae within the healing chambers in TP (Figure 7c) and TP 3DS (Figure 7d), consistent with an intramembranous-like mode of healing.

### 3.3. Histomorphometric Analysis

**Figure 8:**
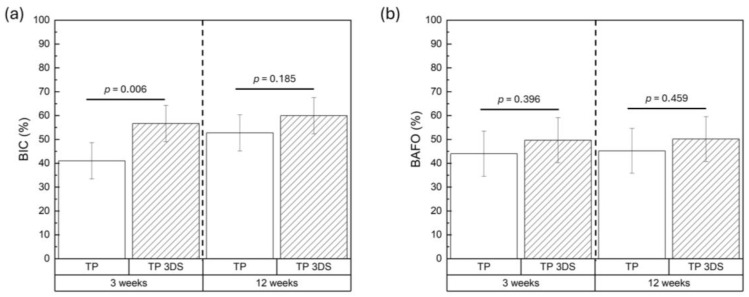
(**a**) BIC and (**b**) BAFO at 3 and 12 weeks post-implantation. *p* < 0.05 is statistically significant.

BIC values were significantly higher 3 weeks post-implantation in the TP 3DS cohort relative to TP (*p* = 0.006, Figure 8a). In contrast, at 12 weeks, no significant differences in BIC were observed between TP and TP 3DS (*p* = 0.185, Figure 8a). On the other hand, BAFO was homogenous between TP and TP 3DS at 3 weeks (*p* = 0.396) and 12 weeks (*p* = 0.459) post-implantation, as shown in Figure 8b.

### 3.4. Nanoindentation Testing

**Figure 9:**
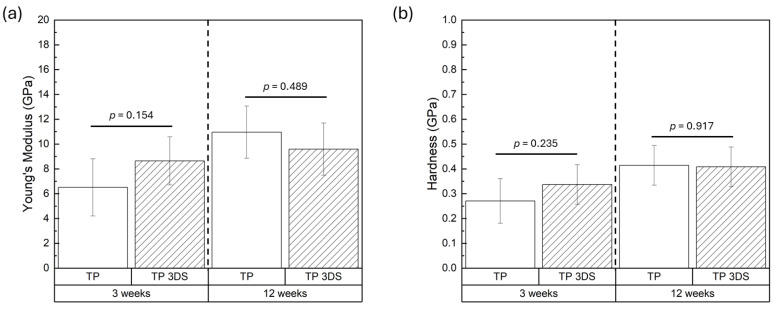
(**a**) Young’s modulus and (**b**) hardness of newly formed bone within the healing chambers at 3 and 12 weeks in vivo. *p* < 0.05 is statistically significant.

Young’s modulus of newly formed bone within healing chambers was statistically similar between TP and TP 3DS at 3 and 12 weeks post-implantation (*p* = 0.154 and *p* = 0.489, respectively, Figure 9a). Similarly, no differences in hardness values were observed between TP and TP 3DS at either 3 (*p* = 0.235) or 12 week (*p* = 0.917) time points, as shown in Figure 9b.

## 4. Discussion

Reduced micromovements have been associated with enhanced bone healing during the early phases of osseointegration [3,11]. Implant stability, which influences resistance to masticatory forces, is determined not only by thread pitch and depth, but also by the thread geometry [29]. In a low-density (Type IV) ovine iliac model, implant micromovements assessed via ISQ measurements indicated that both TP and TP 3DS thread designs exhibited high primary stability immediately post-insertion. ISQ utilizes resonance frequency to ascertain implant stability, and due to its ease of interpretation, it has garnered significant support and clinical implementation [30,31,32]. Previous studies have shown that ISQ value ≥70 is indicative of high implant stability and minimal micromotion, with variations above this threshold generally considered clinically insignificant [26,33]. The interpretations presented in the current study are based on trends observed in the literature pertaining to the use of ISQ as a measure of implant primary stability in an ovine ilium model [34]. However, it is important to note that ISQ values may slightly differ between anatomic locations (bone densities), direction of measurements, site preparation techniques, and/or fitting of smart pegs [35,36,37]. As such, care should be taken when extrapolating pre-clinical ISQ data to clinical settings.

On the other hand, ITV quantitatively reflects bone-to-implant interlocking and is strongly influenced by implant thread design [38]. Notably, both implant groups in the present study exhibited mean ITVs exceeding 60 N⋅cm, a level typically classified as high primary stability in clinical settings (≥30 N⋅cm), with screw-type implants that are inserted into the osteotomy in close contact at the bone–implant interface along the threaded length [39,40,41]. This mechanical interaction enables primary stability in the absence of biological interaction at T = 0 weeks. As such, the mechanical interlocking at this initial time point is mainly impacted by the implant geometry and osteotomy dimensions, which govern the strain exerted on the native bone walls during implant insertion [19]. Theoretically, bone has been described as an elastic material, with a linear relationship between strain and implant stability [42]. However, the implant’s stability diminishes beyond the yield strain of the bone due to excessive microcracking and/or compression-induced necrosis [43]. Therefore, ITV must be carefully considered depending on the quantity/quality of the underlying bone in a clinical context.

Lateral (shear) components of masticatory forces have been shown to exert a more detrimental effect on bone healing than axial (vertical) loads [44,45]. The TP 3DS macrogeometry improved primary stability, demonstrating approximately a ~66% increase in baseline lateral load-bearing capacity compared to TP. Previous studies have indicated that implant stability within the trabecular compartment arises from mechanical interlocking with the surrounding trabeculae [46,47]. The distinctive curved and linear surface features along the entire threaded length of the TP 3DS implants have been shown to function as a mechanical interlocking and friction retention mechanism, which may also help explain the results observed in this study at T = 0 weeks [26]. It is hypothesized that, prior to osseointegration, the combination of curved and straight geometries of the TP 3DS facilitated a more uniform distribution of forces across a larger surface area of underlying bone compared to the TP. However, this hypothesis requires validation in future studies, potentially through finite element analysis (FEA).

Quantitative histological analysis revealed higher BIC values for the TP 3DS implants compared to TP at 3 weeks due to a largely uninterrupted bone–implant interface. Moreover, the presence of cutting flutes along the entire length of the TP 3DS implant permitted a more even distribution of bone chips. These bone chips have the capacity to serve as nucleation sites, which aid in woven bone formation and early osseointegration outcomes [21]. On qualitative analysis of histological sections, an increased presence of woven and lamellar bone was observed in both implant groups. This aligns with the theoretical basis for initial stability, which is primarily achieved through mechanical interlocking between the implant and bone. Over time, however, this stability diminishes due to bone resorption and remodeling under stable conditions [48,49]. In addition, healing chambers were initially filled with blood clots, which later progressed to osteogenic connective tissue.

At 12 weeks, ossification occurred through an intramembranous-like pathway, with no evidence of bone resorption or dieback observed in either implant design. Furthermore, over time in vivo, a significant remodeling area became evident, with voids partially filled by newly formed bone in both groups. No differences in BAFO (indicative of secondary stability) or lateral load parameters were observed at 3 and 12 weeks. This suggests that the biomechanical benefits of the TP 3DS over their TP counterparts at the 0–3-week time interval may diminish at later time points due to the high trabecular bone turnover rate. For example, a previous biomechanical study of trabecular bone showed an ~8-fold increase in remodeling rate compared to cortical bone (26% turnover/year vs. 3% turnover/year, respectively) [50].

Both Young’s modulus and hardness were equivalent between TP and TP 3DS at both time points, potentially owing to the identical surface topographies and biologically inert Ti64 composition. Additionally, we suspect the high turnover rate of trabecular bone relative to cortical bone causes a rapid equivalency in secondary stability as described above. This is corroborated by findings of Marão et al., where differences in bone healing as a result of changes in implant macrogeometries were more noticeable in cortical relative to trabecular bone [3]. For implants targeted at sites with high trabecular bone, such as the Type IV bone utilized in the current study, or in subjects with systemically compromised conditions, bioactive surface coating or nano-topography changes should be analyzed to further expedite bone healing and improve osseointegration outcomes. For example, our prior study compared a nanometer-level textured (nano-hydroxyapatite) implant surfaces with conventional micrometer-level textured (dual acid-etched) implants in pre-clinical swine models of compromised healing—type 2 diabetes mellitus and obesity/metabolic syndrome versus healthy controls [51]. Increased levels of bone formation were recorded for nano-hydroxyapatite-coated implants compared to their dual acid-etched counterparts. This increase in bone formation between the surfaces was more significant in metabolically compromised animals in comparison to the healthy controls [51]. Of note, the nano-textured implants placed in compromised experimental groups exhibited similar levels of bone formation to the micro-textured implants placed in the healthy control group [51]. Thus, the present results and existing literature substantiate the hypothesis that bioactive micro- and nano-meter scale surface modifications may mitigate the adverse effects of O/MS and T2DM, necessitating further pre-clinical studies prior to clinical trials [52,53].

## 5. Conclusions

In a low-density (Type IV) bone model, the TP 3DS implant demonstrated improved performance compared to the conventional TP, as evidenced by an increase in baseline lateral loading capacity and increased BIC during the early stages of osseointegration. These findings indicate that the modified implant configuration of TP 3DS facilitates more favorable biomechanical integration and may promote more rapid and stable bone anchorage under compromised bone quality conditions. Future analyses should delineate the influence of bioactive surface coatings on the primary and secondary stability of TP 3DS implants. Such improvements could have important clinical implications for the success and longevity of implants placed in low bone density regions or in patients presenting with compromised systemic conditions.

## Data Availability

The raw data supporting the conclusions of this article will be made available by the authors on request.
